# Co-existing of HSV1/2 or EBV Infection with the Presence of High-Risk HPV DNA in Cervical Lesions in the Southwest of Iran

**DOI:** 10.31557/APJCP.2020.21.5.1459

**Published:** 2020-05

**Authors:** Negar Joharinia, Sajad Faghihinezhad, Keyvan Seyedi, Ali Farhadi, Seyed Younes Hosseini, Akbar Safaei, Helen Baharampour, Jamal Sarvari

**Affiliations:** 1 *Department of Bacteriology and Virology, School of Medicine, Shiraz University of Medical Sciences, Shiraz, Iran. *; 2 *Diagnostic Laboratory Sciences and Technology Research Center, School of Paramedical Sciences, Shiraz University of Medical Sciences, Shiraz, Iran. *; 3 *Department of Pathology, School of Medicine, Shiraz University of Medical Sciences, Shiraz, Iran. *; 4 *Gastroenterohepatology Research Center, Shiraz University of Medical Sciences, Shiraz, Iran. *

**Keywords:** Human Papilloma Virus, EBV, HSV, co-infection, Cervical lesions

## Abstract

**Objective:**

While the vast majority of the cervical lesions have been attributed to the HPVs, the role of EBV and HSV1/2 as co-factors in the progression of these abnormalities needs more investigation. In this study, we aimed to determine the co-existence of EBV or HSV in cervical lesions infected with high-risk HPVs.

**Methods:**

Totally, 102 formaline-fixed cervical lesions with different pathological grades (LSIL, HSIL, and SCC) were enrolled in this study. DNA was extracted, and its integrity was examined by PCR assay. Two conventional PCRs were performed for the detection of EBV and HSV1/2 genomes in the tissue specimens. Besides, an in-house Real-Time PCR, as well as a nested PCR assays following sequencing, was performed to detect HPV genotypes in EBV or HSV positive samples.

**Results:**

The mean age of the participants was 42.8±13 years. Out of 102 samples, 32% (n=33) were confirmed to be LSIL, 42.2% (n=43) were HSIL, 22.5% (n=23) were SCC and 2.9% (n=3) were adenocarcinoma. EBV genome was detected in 13(12.7%) samples including 2 of LSIL, 8 of HSIL and 3 of SCC. All EBV positive samples harbored high risk HPV types 16,18 and/or 31 co-infections. However, the HSV genome was not found in any of the samples.

**Conclusion:**

Our result revealed that the frequency of EBV infection is higher in HISL than LSIL. Moreover, the amount of HPV load showed an elevated level among co-infected patients, which indicates that EBV might be an enhancing factor of disease progression. In contrast, HSV may not has a role as a co-factor in cervical lesions pathogenesis.

## Introduction

Cervical cancer is the fourth most prevalent, and the fifth lethal cancer amongst women worldwide (Fitzmaurice et al., 2017). The association between high-risk oncogenic papillomaviruses (HPVs) and cervical lesions is well known (Khenchouche et al., 2013; Al-Thawadi et al., 2018). The persistence of viral infections is attributed to a significant part of human cancers worldwide (~12–15%) (Shi et al., 2016). It was also suggested that viral co-existence might also make the progression of cervical lesions faster into cervical cancer. The coexisting of sexually transmitted viruses such as Herpes simplex viruses (HSV1, 2), Cytomegalovirus, Epstein- Barr virus (EBV) and, Human immunodeficiency virus are supposed to enhance the infection or disease progress during HPVs persistence (Staykova et al., 2016). EBV and HSV belong to the Herpesviruses that persistently infect the majority of the adult population. While HSV has not been suggested as a direct tumorigenic agent, HPV, along with EBV is associated with 38% of all infection-related cancers (Shi et al., 2016). EBV was the first recognized human oncovirus which is responsible for different lymphoid and epithelial malignancies (Sarvari et al., 2018; Vranic et al., 2018). Oncogenesis potency of EBV in the cervix may be associated with an increased expression of the latency proteins, LMP-1, and EBNA-2 (Szostek et al., 2009b). Studies showed that EBV could transform the cells via EBV/C3d (the third component of complement, C3) receptor interaction which in turn makes them more permissive/sensitive to other oncogenic viruses infection. Biopsies of the uterine cervix showed that the EBV receptors are mostly expressed on ecto- and endo-cervix regions, therefore, it might be involved in the development of cervical cancer as a “helper” factor (Thoe et al., 1993; Shi et al., 2016). Regarding new reports, the presence of EBV has been documented in other cancers including breast, prostate, oral, and salivary gland carcinomas (Sarvari et al., 2018; Dowran et al., 2019). 

The presence of EBV in cervical cancer and its precursor lesions (cervical intraepithelial neoplasia, CIN) was firstly reported in the early 90s (Turner et al., 1990). Earlier studies have proposed that EBV is associated with the development of cervical cancer. In this regard, Marinho-Dias et al., (2013) reported a 10.1 % prevalence of EBV among cervical lesions. Another study by Santos et al., (2009) showed 21.21% and 64.29% of High Grade Squamous Intraepithelial Lesion (HSIL) and cervical cancer were infected with EBV, respectively.

Moreover, it has been shown that the co-infection of EBV and high-risk HPVs in cervical tissues is more frequent in patients with HSIL in comparison with low-grade lesions (Al-Thawadi et al., 2018). A recent study reported 43.63%. of EBV infection in cervical cancer. They also reported that *EBV* gene expression levels gradually increased from 27% in CIN1 to 35% in CIN2/3 (de Lima et al., 2018). Szostek et al., (2009b) suggested that the co-infection of HPV with EBV could increase the integration possibility of the HPV-16 genome that might contribute to the development of cervical cancer. Besides, Khenchouche et al. found the highest co-infection of HR-HPV and EBV in squamous cell carcinoma cases (SCC) (67%) (Khenchouche et al., 2013). Therefore, they concluded that the HR-HPV and EBV co-infection in SCC might accelerate cervical cancer progression. Also, Kienka et al., (2019) reported that 100% (4/4) of HSIL HPV positive samples were infected with EBV suggesting the importance of their co-infection in developing more advanced cervical disease (Kienka et al., 2019).

HSV1/2 is a human pathogen, mainly associated with primary and recurrent genital infections with no transforming property (Motamedifar et al., 2015). The co-factor role of HSV-2 in cervical lesions progression has been postulated to be due to the induction of mutations throughout the HPV genome (Rawls et al., 1968; Hausen, 1982). Yang et al., (2004) reported that HSV-2 was associated with cervical and oral tumors and their study suggests that HSV is a risk factor in the development of cervical and oral cancers. Also, several studies have shown the co-infection of HSV-2 and HPV-16/18 in various cancers such as bladder, oral and vulvar squamous cell carcinoma (Ahmadi et al., 2017). However, the actual role of HSV-2 in the development of transformed cervical lesions remained a question.

Considering the importance of cervical cancer, and the possible effect of persistent viruses in the progression of the tumor diseases, this study was designed to investigate the prevalence of EBV and HSV1/2 infections in patients with cervical lesions in the southwest of Iran, Shiraz. In this regard, the detection of EBV and HSV1/2 genome was performed in high-risk HPV infected lesions. 

## Materials and Methods

*Study design and samples*

In this cross-sectional study, 102 formalin-fixed paraffin-embedded biopsy (FFPE) samples including 33 ISIL cases with CIN I; 23 HSIL cases with CIN II; 20 HSIL cases with CIN III; 23 cases with SCC and 3 other cases with adenocarcinoma were collected. All the samples were selected from the archive of the department of pathology of Motahari Clinic affiliated to Shiraz University of Medical Sciences, 2014-15. The study was approved by the local Ethics Committee of Shiraz University of Medical Sciences Ethic No: 1398.181). The Hematoxylin and eosin-stained slides were reclassified into different histologic grade of cervix LSIL, HSIL and SCC by the pathologist according to the WHO classification of the tumors for the female genital tract classification system (Nayar and Wilbur, 2015).

*DNA extraction and qualification*

Totally, for all samples, 10 μm-thick sections of FFPE tissue blocks were cut and collected in autoclaved microcentrifuge tubes and then subjected to deparaffinization step as described in a previous study (Mahmoodvand et al., 2017). Genomic DNA was extracted using the QIAamp FFPE Tissue Kit (Qiagen, Hilden, Germany) according to the manufacturer’s instructions. The accuracy of the extraction method was qualified by OD metry using (Nanodrop 1000, Thermo Scientific) and the extracted DNA was stored at -20°C until further analysis.

*Real-time PCR assay for β-globin gene*

In order to assess the quality of the extracted DNA and also quantifying the number of cell copies in every sample; all the extracted DNAs were subjected to an in house Real-Time PCR assay to detect β-globin gene as a housekeeping gene to evaluate the integrity of DNA and reporting HPVgenomic copies in a defined and normalized number of cells. The mean values were expressed as HPV DNA copies/ng DNA. The sequences of primers, PCR reaction components as well as amplification conditions were described previously (Joharinia et al., 2019). 

*EBV PCR amplification*

Specific primers for EBV genome detection were designed by AlleleID software, version 7.0 (Premier Biosoft International, Palo Alto, CA, USA). The sequences of primers targeting the BHRF1 gene region of the EBV are provided in Table 1. The presence of the EBV genome was examined in all β-globin positive samples and those with negative β-globin results were excluded from the study. PCR reaction contained Ampliqon 2x PCR mix red (Ampliqon, Odense, Denmark) and 0.4 µM of each primer in 25 µl final volume. Then amplification was done in the following condition: 10 min initial denaturation at 95°C, 45 cycles of denaturation at 95°C for 45 sec, annealing at 57.6°C for 45 sec, extension at 72°C for 45 sec, and a final extension at 72°C for 10 min. PCR products were then loaded into 2% agarose gel and visualized under UV light. The total DNA was extracted from the lymphoid B-95 cell line (Cell Bank of Tehran Pasture Institute, Tehran, Iran) which harbors episomal EBV genome was used as a positive control in each run.

*PCR detection of HSV1/2*

HSV detection was performed using specific primers (Table 1). PCR reaction components included Ampliqon 2x PCR mix red (Ampliqon, Denmark, Odense) and 0.4 µM of each primer (Table 1) in 25 µl final volume. Amplification was done as follows: 10 min initial denaturation at 95°C, 45 cycles of denaturation at 95°C for 45 sec, annealing at 58°C for 45 sec, extension at 72°C 45sec, and a final extension at 72°C for 10 min. To ensure the reliability of the tests, we also included in each run a positive sample that was confirmed as HSV positive as described before (Motamedifar et al., 2015).

*Quantification of HPV 16 and/or18 genomic copies and determination of other HPV genotypes*

A nested PCR assay using MY09/11 consensus primers followed by GP5+/6+ primers was done for the detection of HPV DNA sequences in all samples. HPV negative samples were excluded from the study and HPV positive samples underwent HPV16 and 18 DNA detection and quantification assessments. In this regard, an in-house duplex TaqManTM based real-Time PCR was performed, as described before (Joharinia et al., 2019). The sequences of primers, PCR reaction components as well as amplification conditions were described previously (Joharinia et al., 2019). In order to determine the genotype of HPV detected in the samples that were tested negative for HPV16 and/or18 DNA, we subjected the 150 bp amplified PCR products of GP5+/6+ primers to a bidirectional Sanger sequencing assay. 

*Statistical analysis*

SPSS 21 software was used for statistical analysis. Chi-Square, T-test and Mann-Whitney tests were used to compare different parameters. P values less than 0.05 were considered to be statistically significant.

## Results

*Demographic characteristics of the participants*

The mean age of the patients was 42.8±13 years and that of the LSIL group was 39±13; HSIL 38.6±11.85; SCC 50.7±11 and ADC 67±7. A statistically significant correlation was observed between older ages and severer lesion grade (P <0.001). Out of 102 samples, 32% (n=33) were confirmed to be LSIL, 42.2% (n=43) were HSIL, 22.5% (n=23) were SCC and 2.9% (n=3) were adenocarcinoma. The mean age of EBV positive patients was 45.9 ±14.8 years.

*EBV PCR result*

One sample with a negative β-globin PCR result was excluded from the study and EBV PCR assay was performed for 101 positive β-globin samples. EBV genome was detected in 13/101 samples (12.7%) including, 2 samples with LSIL, 8 with HSIL and 3 with SCC (Table 2). The frequency of EBV infection was not significantly different among studied groups(p>0.05).

*HSV detection PCR result*

In the present study, none of the 101 samples was positive for the presence of HSV DNA in all grades of cervical lesions (Table 2).

*HPV detection and viral genomic copies quantification*

Our results showed that all of the EBV positive samples had co-infection with high-risk HPVs including types 16, 18 and 31 (Table 2). Analysis of the results showed that 3 samples (23.1%) had HPV 16, 1 (7.7%) had HPV18, 1 (7.7%) had HPV 31 and 8 (61.5%) had HPV 16 / 18 co-infection with *EBV* genome. The HPV genomic copies results showed that the mean of HPV type 16 and 18 genomic copies in EBV positive samples were 6.4 with 95%CI (5.8-7.9) and 5 with 95%CI (4.58-5.4) copies of the virus per cell, respectively. These results also indicated that although the mean of HPV-16 genomic copies in EBV/HPV co-infected samples was higher than those HPV16 mono-infected samples (6.05) copies of HPV16 with 95%CI (5.7-6.3) per cell, it was not statistically significant (p-value= 0.4). Similarly, no statistically significant difference (p-value=0.7) was found between genomic copies of HPV18 mono-infected [mean of 4.9 copies of HPV18 with 95%CI(4.7-5.1) per cell]patients in comparison to EBV/ HPV co-infected ones. The co-infection of EBV and HPV was detected significantly more frequently in HSIL lesions and SCC groups than in LSIL (p-value =0.044). 

**Table 1:** The Primer Sequences Used for PCR Tests

Oligonucleotide	Sequence	Product size
EBV F	5′- TACTCCTTACTATGTTGTG-3′	295 bp
EBV R	5′- CCTTGCCTAATATCCTAC-3′	
HSV F	5'- CAG TAC GGC CCC GAG TTC GTG A-3'	465 bp
HSV R	5'- TTG TAG TGG GCG TGG TAG AT-3'	

**Table 2:** Frequency of HSV, EBV and EBV/HPVs Co-Infection in Different Grades of Cervical Lesions

Virus	HSV	HPV	EBV	EBV-HPV co-infection	EBV/HPV 16	EBV/HPV18	EBV/HPV16&18	EBV/HPV31
Frequency	0/101	91/101 (90%)	13/101 (12.8%)	13/101 (12.8%)	3/13 (23%)	1/13 (7.60%)	8/13 (61.50%)	1/13 (7.60%)
LSIL	0	26/91 (28.6%)	2/13 (15%)	2/2 (100%)	0	1/2 (50 %)	1/2 (50 %)	0
HSIL	0	39/91 (42.8%)	8/13 (61%)	8/8 (100%)	2/8 (25%)	0	6/8 (75%)	0
SCC	0	23/91 (25.2%)	3/13 (23%)	3/3 (100%)	1/3 (33.30%)	0	1/3 (33.30%)	1/3 (33.30%)
ADC	0	3/91 (3.2%)	0	0	0	0	0	0

## Discussion

The latency period between HR-HPV infection and cervical cancer progression suggests the involvement of other etiologic agents in malignancy progression. In this regard, the frequent detection of EBV in high-grade cervical lesions, suggests that EBV could be a cofactor of HPV-associated cervical carcinogenesis (Szostek et al., 2009b). 

In this study, we attempted to find a correlation between the presence of EBV and HSV amongst the HPV-positive cervical lesions. The results of our study showed that the frequency of EBV in cervical samples was 12.7% (13 out of 101 samples). In the same line with our results, the prevalence of EBV infection in cervical samples reported by Zhang et al., (1994) was 15.6% (15/93). Similarly, a study from Poland reported an EBV co-existence among 14% HPV positive specimens (Szostek et al., 2009a). However, the study from Bulgaria showed that just 9.6% of all specimens were positive for EBV and HPV simultaneously (Staykova et al., 2016). Also, a higher prevalence of EBV coexisting with HPV reported from India (20%) (Silver et al., 2011), Thailand (32%) (Aromseree et al., 2015) and Syria (34%) (Al-Thawadi et al., 2018), demonstrating a more important role of EBV as a cofactor. 

Our results also showed that the EBV genome was mostly found in HSIL (CINII and CIN III) which may indicate the importance of EBV presence in the progression of lesions. In accordance, it has been reported that the EBV genome was more frequent in HSIL than LSIL lesions (Sixbey et al., 1986; Landers et al., 1993). Furthermore, Silver et al. showed that women with LSIL or higher-grade of cervical lesions were almost 4 times more likely to be EBV positive than those without the disease (Silver et al., 2011). Also, EBV infection in HSIL was significantly more prevalent compared to LSIL (Aromseree et al., 2015). Szostek et al., (2009a) reported that EBV DNA was more frequent in cervical cancer tissue specimens than LSIL and HSIL. Furthermore, simultaneous co-infection of HPV and EBV was associated with more advanced HSIL cervical lesions, as all HPV positive samples with HSIL lesion harbored EBV DNA (Kienka et al., 2019). Khenchouche et al., (2013) showed that the highest co-infection of HR-HPV and EBV was found in squamous cell carcinoma cases (67%). Data from Szostek et al., (2009b) research showed that the presence of EBV DNA was more frequent in cervical cancer tissues than LSIL and HSIL. However, in our study in HSIL but not cancerous samples the prevalence of EBV was significant. 

There are several suggestions regarding the effect of EBV/HPV co-infection on the outcome of the cervical lesion, Al-Thawadi et al., (2018) reported the co-expression of LMP1 and E6 genes of EBV and high-risk HPVs to be an important factor associated with squamous cell carcinomas and overexpression of diffused Id-1, which is an important regulator of cell invasion and metastasis. These data indicated that EBV and HPVs co-infection in cervical cancer is associated with a more aggressive cancer phenotype. During HPV infection, inflammatory signals are detected in cervical lesions and EBV-infected B-cells attracted to such inflammatory lesion; which in turn perform as a co-factor in the progression of cervical lesions (Meckes et al., 2010; Fernandes et al., 2015). EBV may also alter the immune response to HPV-transformed cells by producing the viral BCRF1 gene product, an interleukin-10 homolog which modulates the HPV related tumor environment (Meckes et al., 2013; Polz-Dacewicz et al., 2016). Moreover, data of Zoster et al., (2009b) suggested that co-infection with EBV may facilitate the integration of the HPV-16 genome and subsequently the development of cervical cancer. Also, two studies reported that HPV/EBV co-infection correlated with a 5 to 7-fold increased likelihood of HPV integration into the host genome and enhanced genomic instability of HPV-infected cervical epithelial cells (Szostek et al., 2009c; Kahla et al., 2012). Furthermore, Aromseree et al. declared that HPV–EBV co-infection was more common in episomal HR- HPV and EBV may have a role in carcinogenesis even in the absence of integrated HPV-DNA (Aromseree et al., 2015). Another theory that supports cancer progression by EBV is the possible EBNA1 roles which can lead to the transformation of cells through down-regulation of apoptosis and triggering DNA repair (Frappier, 2011; Kahla et al., 2012). The result of HPV genomic quantification in tissue specimens infected with both HPV and EBV showed that the average HPV 16 genomic copies in mono-HPV infections were lower than those co-infected with EBV and HPV. This finding indicates that EBV might have an effect on cellular pathways and interferes with HPV replication. Higher copy numbers of HPV-16 in EBV/HPV co-infected lesions could be determined as a bad prognosis in cervical cancer progression. 

Regarding the frequency of HSV infection in cervical lesions, our results did not show the presence of HSV1/2 genome in cervical lesions and cancerous tissues. Similar to our results, another report from Iran also showed no prevalence of HSV in cervical cancer tissues using real-time PCR assay (Farivar et al., 2012). Ahmadi et al., (2017) also showed that only one out of 45 patients (2.22%) was infected with HSV-2. In contrast, in a study carried out in Bulgaria, the prevalence of HSV infection was 30.8% in HPV infected lesions (Staykova et al., 2016). Besides, Thanh et al. reported that HSV-2 could not be a cofactor to HPV in the etiology of cervical malignancies(Tran-Thanh et al., 2003). The low prevalence of HSV-2 is probably due to the fact that cervical sampling is not appropriate for this type of study because of the characteristics of viral biology related to neurovirulence (Rocha et al., 2012). 

In conclusion, the results of this study showed that the frequency of EBV infection is higher in HSIL (CINII and CIN III) grade than LSIL. Moreover, higher HPV genomic copies was found in samples infected with both HPV and EBV than those only infected with HPV. Therefore, the co-infection of EBV and HPV might influence the progression of cervical lesions towards higher pathological grades. Also, our findings showed that there was no association between HSV infection and different grades of cervical lesions. Further investigation is required to clarify the role of EBV and HSV infection in cervical cancer. 
